# Endogenous Mechanisms of Neuroprotection: To Boost or Not to Be

**DOI:** 10.3390/cells10020370

**Published:** 2021-02-10

**Authors:** Sara Marmolejo-Martínez-Artesero, Caty Casas, David Romeo-Guitart

**Affiliations:** 1 Department of Cell Biology, Physiology and Immunology, Institut de Neurociències (INc), Universitat Autònoma de Barcelona (UAB), Bellaterra, 08193 Barcelona, Spain; Sara.Marmolejo@uab.cat; 2 Laboratory “Hormonal Regulation of Brain Development and Functions”—Team 8, Institut Necker Enfants-Malades (INEM), INSERM U1151, Université Paris Descartes, Sorbonne Paris Cité, 75015 Paris, France

**Keywords:** autophagy, cellular resilience, endogenous mechanisms, neuroprotection, neuronal survival, unfolded protein response

## Abstract

Postmitotic cells, like neurons, must live through a lifetime. For this reason, organisms/cells have evolved with self-repair mechanisms that allow them to have a long life. The discovery workflow of neuroprotectors during the last years has focused on blocking the pathophysiological mechanisms that lead to neuronal loss in neurodegeneration. Unfortunately, only a few strategies from these studies were able to slow down or prevent neurodegeneration. There is compelling evidence demonstrating that endorsing the self-healing mechanisms that organisms/cells endogenously have, commonly referred to as cellular resilience, can arm neurons and promote their self-healing. Although enhancing these mechanisms has not yet received sufficient attention, these pathways open up new therapeutic avenues to prevent neuronal death and ameliorate neurodegeneration. Here, we highlight the main endogenous mechanisms of protection and describe their role in promoting neuron survival during neurodegeneration.

## 1. Neurodegenerative Processes

With increasing life expectancy in developed countries, the frequency of neurodegenerative diseases such as Alzheimer’s disease (AD), Parkinson’s disease (PD) or Huntington’s disease (HD), or age-related decline of our nervous system performance, are likely to increase. Although there are several lines of evidence indicating that these pathologies have neuronal, astroglial, and microglial components, the decline in daily functions is caused by a progressive neuronal loss. Due to their low turnover, neurons are postmitotic cells that must live for a lifetime. For this reason, they need a powerful intrinsic protective machinery to cope with external and internal insults, which will cause their demise. These external/internal hazards are traumatic injuries or excitotoxic compounds, reactive oxygen species (ROS), protein aggregates, and other toxic molecules. Fortunately, cells have intrinsic machinery that blocks death by activating resilience mechanisms or promoting regeneration pathways. While young neurons have proper functioning of these self-healing protective mechanisms, aging disturbs them, leaving the neurons unprotected. In the same direction, dysfunctionality on these self-healing mechanisms has also been described in neurodegenerative diseases.

During the last decades, enormous efforts have been invested in obtaining novel and effective neuroprotective therapies. However, they are intended to target pathophysiological mechanisms, which at the end turns into an acceleration of neuronal demise. Therefore, why not boost the mechanisms that neurons have naturally to obtain an effective neuroprotective approach?

This protective network is driven by the crosstalk of different cellular processes (i.e., unfolded protein response (UPR), autophagy, etc.), but they converge into the same process: allowing the cell to adapt to stress and survive [1,2,3]. Recently, we have considered a novel rationale to discover neuroprotectants: decipher what molecular mechanisms neurons engage after two different nerve injuries with opposite phenotypes, survival or death, which share similarities with health and neurodegeneration/aging. To do so, we used two in vivo-based peripheral nerve injury models that mimic functionality or dysfunctionality of the endogenous mechanisms of protection. They provoke either motoneuron (MN) death (root avulsion (RA)) or survival (distal axotomy (DA)), depending on the soma–injury distances [2]. With the help of these models and using a Systems Biology-based approach, we confirmed that the death of MNs after RA shares similarities with the neuronal loss observed in neurodegenerative diseases, and we also described which mechanisms are used by MNs to survive after nerve injury [2]. Degenerative processes are apoptosis, necrosis, anoikis, endoplasmic reticulum (ER) stress, nucleolar stress, cytoskeletal rearrangements, and mitochondrial dysfunction, while the drivers of survival are: a correct UPR, the heat-shock response, the autophagic pathway, the ubiquitin-proteasome system, the chaperone systems, the ER-associated degradation machinery and the antioxidant defense (Table 1). Interestingly, all these mechanisms have been separately described years ago and referred to as pre-conditioning injuries (see below).

We have demonstrated that boosting these endogenous mechanisms of neuroprotection through pharmacological treatment allows MN to survive in different pro-death scenarios, ranging from different species to different stages of development [23,54,55].

## 2. First Evidence of Endogenous Mechanisms: Pre-Conditioning

The phenotypic effects of endogenous mechanisms of protection were described 40 years ago. In 1986. Murry et al. described that sublethal physiological stress, also known as preconditioning injury, enhances tissue recovery in the heart [56]. From here, these healing mechanisms were also observed in the brain and spinal cord (SC) [57]. For example, these cellular responses are observed after nerve injury or during heart regeneration, where the production of ROS or extracellular vesicles respectively, drives functional recovery [58,59,60]. Surprisingly, the pre-conditioning of a specific organ exerts protection to others from injury [61]. Several specific effectors are responsible for these effects. After preconditioning injury, the production of different mediators (nitric oxide or ROS) will activate the signaling pathways phosphatidylinositol 3-kinase (PI3K)/Protein kinase B (AKT), Protein kinase C (PKC), and other signaling pathways that will modulate transcription factors such as Hypoxia-inducible factor 1-alpha (Hif1-α) or NF-κB. These will result in the production of nitric oxide synthases (iNOS), heat-shock proteins (HSPs), and cyclooxygenase-2 (COX-2), which are described as "end effectors", and will promote the protective effect within the tissue against future insults [61]. Together, these studies suggest that organisms/cells have endogenous protective mechanisms, and boosting them may be an effective therapeutic strategy.

## 3. Endogenous Mechanisms of Neuroprotection

### 3.1. Fine-Tuning Autophagy

Neurons require continuous recycling of intracellular materials to maintain homeostasis. Macro-autophagy, hereafter referred to as autophagy, is a highly coordinated molecular network in eukaryotic cells that pursues to recycle cytoplasmic content through lysosomal degradation. Although this degradation mechanism was initially observed only under starvation, recent studies showed that cells have a basal level of autophagy to regulate protein homeostasis. These basal levels are essential for axonal maintenance and survival of neurons under normal conditions [62,63]. A functional autophagic flux is a process highly coordinated by different autophagy-related (*ATG*) genes, kinases, and other regulatory proteins. They all work together to orchestrate the correct initiation, nucleation, elongation, closure, and fusion of autophagosomes with lysosomes to degrade the cytosolic load [64]. A reduced flow of autophagy is observed in the hippocampus during aging, while the reestablishment of its levels facilitates the formation of new memories [65]. Impaired or dysfunctional autophagy in neurons is associated with neurodegeneration, while activation of autophagy produces neuroprotection [5,54]. Alterations in the proteins related to the initial and elongation phases have been observed in amyotrophic lateral sclerosis (ALS) [66,67], and inducers of autophagy, such as rapamycin, exert neuroprotection after cerebral ischemia, traumatic brain injury (TBI), and AD [68,69,70]. The neuron-specific knockout (KO) of ATG5 or ATG7 causes neurodegeneration, accumulation of cytoplasmic inclusion bodies, and death of neurons [62,71], while their overexpression is beneficial in a model of PD [4]. Finally, p62, which manages the charge in the autophagosome and plays a key role in the late stages of autophagosome formation, is neuroprotective in fly models characterized by protein aggregates, which is a hallmark of neurodegenerative diseases [6].

Several studies have shown the accumulation of autophagosomes and autolysosomes during neurodegeneration, suggesting that autophagy is overactivated and may trigger neuronal death. Aberrant accumulation of autophagic processes within the cytoplasm may be caused by lysosomal dysfunction, rather than overactivated autophagy [72]. Autophagy is properly initiated after TBI, but autophagosomes are not eliminated due to lysosomal dysfunction, leading to unresolved autophagy that promotes neuronal death [73]. These non-functional lysosomal pathways are also seen after a spinal cord injury (SCI), hampering functional recovery [74]. A similar blockage in the clearance of autophagosomes is also described in neurodegenerative diseases (i.e., the human brains of AD) [75]. The integration of all this evidence suggests that enhancing the resolution of autophagy can yield protection. Platt recently highlighted the therapeutic avenue of improving the function of lysosomal proteins to prevent neurodegeneration [76]. The overexpression of transcription factor EB (TFEB), which modulates a transcriptional network essential for lysosome biogenesis and function, has promoted neuroprotective effects in a rat model of PD [7] and an AD mice model [8].

The induction of autophagy is not as good as we would like. Although it is a canonical protective mechanism, its machinery or overactivation can facilitate cell death [77,78]. Inhibition of autophagy after exposure to human prions reduces neuronal damage, indicating that induction of autophagy also drives death [79], and reduced autophagy initiation promotes functional recovery after SC hemisection, prevents apoptosis, and reduces pyramidal death after ischemia in neonatal and adult mice [80,81,82]. If we focus on axotomized neurons, blocking autophagy is neuroprotective for the rubrospinal ones [80], while an increase in the level of ATG5 protects spinal MNs [5]. Adding controversy, cancer cells treated with chemotherapy activate autophagy to overcome treatment-induced apoptotic death, while MN-dependent autophagy inhibits apoptosis [54]. Besides, ATGs also trigger neuronal death. ATG5 loses its pro-autophagic capabilities when cleaved, moving its activity towards the induction of cell death [83,84,85]. Beclin1 has anti-apoptotic effects under normal conditions, but its cleavage at the *C*-terminus sensitizes the cells to apoptotic signals [9]. Therefore, there is a crosstalk between both cellular processes, and the cells can redirect them to increase their chances of survival to cope with the insult [83].

So, what is important for neuroprotection? Boosting or blocking autophagy? Fine-tuning is the answer [86]. Induction of a fine-tuned autophagy yields beneficial effects by: (i) removing non-functional proteins/organelles, (ii) allowing the cell to readapt to the new situation, and (iii) degrading harmful effects such as inflammation or apoptotic inducers [87,88], which mediate neuronal demise. However, this autophagy must be activated in a very specific window of time, avoiding excessive degradation that provokes cell death.

Lastly, autophagy also has non-canonical/degradative functions, such as the modulation of the inflammatory response, the formation of new memories [65], the maintenance of synaptic homeostasis [89], and the transport of cargo within the cell [90]. So, complete blocking of it will lead to irreversible damage to the nervous system and/or neurons.

### 3.2. Tackling the Sexy Part of Unfolded Protein Response

Neurons are extremely sensitive to misfolded proteins and aggregates. The ER is responsible for cellular proteostasis, which is the synthesis, folding, and sorting of proteins. Any alteration in its fitness will lead to the accumulation of misfolded proteins, inducing ER stress and activating the ER-overload response (ERO), the ER-associated degradation (ERAD) pathways, or the UPR, which is a highly conserved cellular response. Alterations in the distribution and morphology of the ER and the UPR have been observed in neurodegenerative diseases [91,92,93] and when the neuron is isolated after a nerve injury [16,94]. Binding immunoglobulin protein (BIP), also known as GRP78, is an ER-resident chaperone that is the main sensor of the UPR. In the inactive state, BIP remains bound to the three major UPR effectors: the RNA-activated protein kinase-like ER kinase (PERK) which induces C/EBP homologous protein (CHOP), the inositol-requiring protein-1 alpha (IRE1α), which splices X-box binding protein 1 (Xbp1) mRNA, and the activating transcription factor-6 alpha (ATF6) [95,96]. When BIP detects misfolded proteins, these transducers are activated and drive changes in the gene expression of specific proteins (i.e., chaperones, transcription factors) with the aim of increasing the cell’s ability to correctly fold proteins by modulating gene expression, enhancing the clearance of misfolded proteins’ clearance, or inhibiting protein synthesis, allowing the cell to adapt to the stress and survive [97]. As a proof of concept, BIP overexpression in dopamine neurons increases their survival, while its downregulation induces the death of nigral dopamine neurons [10]. Besides, BIP ^+/−^ mice show accelerated propagation of prion pathogenesis [98]. Overall, UPR modulation may exert protective effects on neurodegeneration [94], as reviewed recently by our group [99].

UPR activation is an early event in neurodegenerative diseases, and its precise modulation has beneficial effects on pathology progression [100,101]. Although UPR may act as an endogenous mechanism of cell protection, its (over)activation promotes apoptosis [102] (i.e., the PERK axis has pro- or anti-apoptotic capabilities [91]). Besides, recent evidence suggests that different perturbations of the ER will activate differentially the 3 branches of the UPR, indicating that the coordinated co-activation of them is not always present. Therefore, the cell has a specific program to respond to a specific insult. For instance, CHOP blockage or Xbp1 overexpression increases neuron survival after nerve injury, indicating that each branch has different roles in neuron death [16].

The early activation of PERK after brain injury exerts neuroprotection, while the sustained signaling through this pathway exacerbates cell loss [11]. Overexpression or pharmacological PERK activation reduces Tau pathology [12], while averting its sustained activation diminishes neuronal death [13] and improves age-related memory decline [14]. The inhibition of PERK in astrocytes delays neuronal loss in a prion-disease in vivo model. Interestingly, PERK activation in astrocytes disturbs the secretome, altering its synaptogenic function and causing synaptic loss [15]. The same authors described that the main downstream mechanisms involved in this detrimental effect of PERK are the extracellular matrix-cell adhesion pathways, which crosslink the UPR with the anoikis (see below, Section 3.4). Activating transcription factor 5 (ATF5) levels are directly dependent on the activation of PERK/eukaryotic translation initiation factor 2a (eIF2a). ATF5 has been directly linked to those neurons that are more resilient to death in human epilepsy [26]. However, the subsequent consequences of these effects are not as clear. ATF5 induces the expression of two anti-apoptotic effectors (see below), B-cell lymphoma 2 (Bcl-2) and induced myeloid leukemia cell differentiation protein (Mcl-1) [103], which will inhibit apoptosis. ATF5 also modulates the mechanistic target of rapamycin (mTOR) in non-neuronal tissues, which is the main modulator of autophagy, interrelating UPR and autophagy.

Activation of IRE1α ameliorates liver failure [17], and its downstream effector Xpb1 promotes cardiac protection [18], neuroprotection in AD, in PD, and after stroke [19,20,21]. Strikingly, a study in diabetic and ischemia-induced retinopathy showed that the protective effects of UPR are mediated by Xbp1 [22]. Nonetheless, chronic activation of the IRE1α branch will lead to phosphorylation of the tumor necrosis factor-a (TNF-α) receptor-associated factor 2 (TRAF2), triggering apoptotic cell death in different ways [104,105,106]. Ectopic overexpression of Ire1α will lead to an autophagy-dependent neuronal death in a PD *Drosophila* model [107]. Therefore, an adjusted modulation of IRE1α-Xbp1 during a specific window may exert protection [108].

We recently described that NeuroHeal pharmacological treatment or sirtuin1 (SIRT1) overexpression induces survival of MN after nerve injury, and increases the presence of cleaved ATF6 while reducing IRE1α phosphorylation [23]. Pharmacological activation of ATF6 induces protection in different ischemia models by activating proteostasis [24], and the blockage of this transcription factor has deleterious effects. In detail, ATF6 modulates antioxidant-response-related proteins’ expression, modulating the ROS hormesis [109]. Forced expression of ATF6 improves functional outcome after stroke, and the authors suggest that this effect may be mediated by the induction of autophagy [25].

So, what is therapeutically interesting, activate, or attenuate UPR? The activation of specific branches of the UPR is the key point. Precise activation of the UPR can promote protective effects by helping the cell to restore proteostasis. Nonetheless, this concept should be taken with caution because if stress persists and proteostasis is not restored, the UPR triggers neuronal apoptosis which is mediated by the PERK or IRE1α branch [110]. In addition, UPR is also connected with autophagy and vice versa. BIP mediates the autophagic response, promoting neuronal survival [111]. Lastly, the 3 branches of UPR modulate the transcription of ATG’s [112], suggesting an intricate link between both cellular processes.

### 3.3. “Not Today” Apoptosis

Apoptosis is a caspase-dependent programmed cell death (PCD) that maintains the integrity of the cell plasma membrane and the organelles [113]. Its dysregulation is the cause of many cancers, neurodegenerative or inflammatory pathologies. Caspases-induced death is a highly controlled process that needs the coordinated actuation of several players to cause the final cell death [114]. Apoptosis-like death hallmarks are found in Amyotrophic lateral sclerosis (ALS) mice models, AD, or PD, although is unclear if it is the final executor of neuronal demise [115]. During evolution, cells have developed several mechanisms to prevent their death when it is unneeded or to avoid a premature PCD. Cells only trigger an efficient apoptotic death when the balance between pro- or anti-apoptosis machinery pushes them towards death. Based on our in vivo models, we observed that RA induces apoptotic pathways but also anti-apoptotic ones, and their balance leads to an alternative and unknown death that is not the classical apoptosis [2]. Last publications in the field suggest that caspases also act by remodeling the nervous system without promoting cell death [116], and their activity depends on its subcellular position. Therefore, the active forms of caspases that are found in neurodegenerative tissues can have a non-death-related role and the final neuron death is through other fatal mechanisms.

Apoptosis can be impeded by the anti-apoptotic pathways, which are driven by three protein families: FLICE-inhibitory proteins, Bcl-2, and Inhibitors of Apoptosis Proteins (IAPs). IAPs exert neuroprotection in an ischemia model [27] or avoid the death of MNs after nerve injury during neonatal stages [28]. IAPs are proposed to be responsible for the blockage of neuronal death after axotomy during adulthood [29]. In the same direction, a post-translational modification of the X-linked–IAP (XIAP), which blocks its anti-caspase 3 function, has been described as a contributor to the PD pathogenesis [117].

Ischemic preconditioning, which partially reduces the detrimental effects of ischemia, acts through IAPs, and enables cells to survive after caspase cascade activation [30]. IAPs also mediate the pro-survival effect of glial cell-derived neurotrophic factor (GDNF) on MNs after neonatal axotomy [28]. Other molecular pathways that avoid cell death by modulating pro-apoptotic proteins are extracellular signal-regulated kinases (ERK) and AKT. In this sense, the AKT pathway has been described as a pro-survival player by blocking apoptosis [31]. AKT inhibits the apoptosis inducer p53 by promoting its degradation and therefore blocks its pro-apoptotic abilities [32,33,34]. Otherwise, caspases are able to inhibit AKT by its cleavage, which points out a fine-tuned modulation of cell survival and death [118]. On the other hand, AKT activity phosphorylates the Forkhead box protein O (FOXO) transcription factors. They are related to apoptosis [119] and their modification turns on an increased cell survival [35]. AKT-dependent phosphorylation of FOXOs avoids its entrance to the nucleus, averting the induction of pro-apoptotic genes such as the Bcl-2-interacting mediator of cell death (BIM) or Bcl-2 nineteen-kilodalton-interacting protein 3 (Bnip3) [119,120,121]. On the other hand, post-transductional modifications of FOXO fine-tune their transcriptional network within the cell, moving it towards autophagy induction instead of apoptosis [54,121,122,123]. Therefore, specific modulation of the FOXO family is a novel avenue to promote neuronal survival by inhibiting apoptosis [54,124].

Finally, neuronal activity is also a promoter of anti-apoptosis by NMDA-dependent anti-apoptotic genes’ upregulation [125,126]. Some of these upregulated genes allow mitochondria to become more resistant to stress [126], helping the cell to survive the insult.

### 3.4. Reattaching by Anti-Anoikis

Interaction between the cell and extracellular matrix (ECM) is essential for its correct functional integration within the tissue. When this crosstalk is averted, the cell dies through a PCD termed anoikis, which shares pathways with apoptosis. Interestingly, the breakdown of the intrinsic anoikis programs confers malignancy to tumoral cells, giving them enough cellular resilience to escape and reattach onto other tissues without dying [127,128]. The major effectors of these interactions are the integrin proteins, which are formed by the combination of α and β subunits. This combination will determine the ligand specificity and intracellular signaling. ECM signals are transmitted to neurons through integrins, being essential for cell shape, survival, motility, proliferation, development, neuronal connectivity, and synaptic plasticity [129]. Integrins are also important for the intracellular signaling of the growth factors [130], which are well-known modulators of neuronal survival by blocking pro-death mechanisms. β1 integrin subunit is essential for cell-ECM interaction, and its blockage is sufficient to trigger anoikis [36] and neuronal apoptosis [131]. Besides, the intracellular signaling of this subunit is related to the survival of the retinal ganglion cells [132], and their defects are present in neurodegenerative disorders [133].

Nonetheless, cells have developed anti-anoikis subroutines to counteract death, which are initiated by tyrosine kinases, small GTPases [128], NF-κB [134], PI3K/AKT, proto-oncogene tyrosine-protein kinase (Src) or ERK axes, and by autophagy [135,136]. NF-κB modulates anti-anoikis by the triggering of anti-apoptotic proteins such as Bcl-2 and IAP-1 [135], meanwhile PI3K/AKT’s role in cell survival is widely documented and contributes to the survival of differentiated cells [36,37]. ECM detachment also induces autophagy, which is a self-protective mechanism leading to bypass apoptosis [135]. These pieces of evidence suggest again an intricate network between self-protective mechanisms.

Anoikis is also present in neuronal death after TBI due to the increase of matrix metalloproteinase (MMP) that destroys ECM proteins [137]. MMPs expression and levels are modified after neurotrauma, and they have different roles in axonal degeneration, glial scar formation, and synaptic remodeling. Regarding neuronal survival, the inhibition of MMP9 exerts protective effects in cerebral ischemia by the reduction of laminin degradation [38]. MMPs are also implicated in neurodegeneration [138]. Recent studies described that the inhibition of MMP9 has protective effects in the motor unit from an ALS mice model [39,40] and in AD models [41]. Therefore, treatments to inhibit specific MMPs will indirectly maintain the anti-anoikis program within neurons facilitating its survival.

### 3.5. Cytoskeleton and Motor Transporters

The neuronal cytoskeleton is composed of three different structural complexes: microtubules (MTs), intermediate filaments (IF), and actin microfilaments. They have different cellular functions: MT regulates neurite and dendrite dynamics [139], actin is in charge of cell morphology [140], and IF drives mechanical and stability to cytoskeleton structure [141]. Defects in the structural complexes are observed in neurodegenerative diseases, in peripheral neuropathies, in synaptic dysfunction, and lead to mature spine loss [141,142,143,144,145,146].

The dynamic of MTs is a highly controlled process, and its imbalance can carry out devastating consequences for neuron survival or axon performance [142], while its stabilization blocks neuronal death [147] and accelerates axonal growth in the central nervous system [148]. More in detail, cytoskeletal structures are the railways, while the kinesin and dynein motor proteins are the trains that transfer the cargo by anterograde or retrograde transport, respectively. Therefore, motor complexes are also essential for the survival of neurons. The kinesin family is formed by the kinesin-1 (historically named KIF5c) and the kinesin-3 (KIF1A, KIF1Bα, and KIF1Bβ) members [149]. KIF5c is enriched in MNs [150], and its genetic ablation is linked with MN diseases and paralysis [149,151]. It has been recently implied in the pathogenesis of ALS [152]. The impairment of its interaction with MTs leads to axonal degeneration and subsequent neuronal death [153]. KIF5c disruption leads to mitochondrial dynamics disorders, which results in neuronal survival or death depending on the stimuli. Moreover, KIF5c fine-tunes mitochondrial function, turning into cellular health (see below, Section 3.6.) [42], and its modulation may promote neuroprotection. Protein aggregates, such as amyloid-β, have a detrimental effect on KIF5a stability, leading to impaired mitochondrial movement and well function [154]. 

The retrograde proteins also exert neuroprotection. They are the dynein’s and are multiprotein complexes formed by different proteins, with the p150glue (dynactin1/DCNT1) being the most abundant subunits. A dysfunctional dynactin subunit 1 (DCTN1) has been used as an ALS mice model, and its mutation causes a defective axonal transport that leads to an ALS-like phenotype in mice [155,156]. The KO mice show age-dependent MN death, which is accompanied by an autophagy blockage [157]. DCTN1 has a clear role in autophagic vacuoles transport within the neuronal body, and its disturbance causes amphisome accumulation in distal axons, leading to AD-like phenotype [158]. The dynein adaptor Rab-interacting lysosomal protein (RILP) plays a crucial role in autophagosome biogenesis, transport, and its inhibition causes autophagic processes accumulation [44]. Altogether, it has been observed that MT dysfunction, together with kinesin and dynein aberrant localization, leads to lysosomal dysfunction, which provokes autophagosome accumulation and presynaptic dystrophy in AD [159]. The overexpression of DCTN1 in osteoclast prevents apoptotic death, suggesting that motor proteins also have a role in avoiding cellular death in other cell types and tissues [43].

In summary, a reduction in axonal transport is present in many neurodegenerative diseases and after nervous system injury. That defect will result in alterations in MT structure and/or molecular motors required for axonal transport [5]. Proper axonal transport is critical for the normal functioning of neurons, and impairments in this process contribute to neuronal demise. Boosting the transport machinery of the cell, either by stabilizing the cytoskeleton or enhancing motor protein levels/activity, has demonstrated to be neuroprotective by re-establishing a correct autophagy flux within the neuron [5].

### 3.6. Mitochondrial Well-Function

Neurons function depends on energy and calcium (Ca^2+^) balance, so mitochondria performance is crucial for them. Mitochondria are not static organelles. They change shape, size, number, or localization inside the cell and have the ability to fuse or divide by fission to adapt to cellular demand. They produce energy through the tricarboxylic acid cycle (TCA) and oxidative phosphorylation (OXPHOS) via the electron transport chain (ETC). OXPHOS activation will lead to ROS, which has a wide range of functions (differentiation, autophagy, immune response) at physiological levels [160] and axonal regeneration [60]. Nonetheless, at supra-physiological levels, ROS are harmful because they cause damage to lipids, DNA, and proteins. These alterations have been linked to neurodegenerative diseases, SCI, and TBI. Mitochondria also acts as a core regulator of neuronal survival through their involvement in pathways that modulate neuronal death.

Mitochondria are transported around the cell by the cytoskeleton, motor proteins, and appropriate adaptors. In neurons, they are mainly trafficked on MTs by the adaptors Miro and Milton/trafficking kinesin-binding protein 1 (TRAK) proteins [161]. These mitochondria movements within neurons are essential to maintain optimal fitness within the synapses, producing energy, buffering Ca^2+^, etc. [162]. Mitochondria often localize close to ER, forming mitochondria-associated ER membranes, or mitochondria-associated membranes (MAMs). These membrane microdomains are reversible tethers that co-regulate and influence a variety of cellular processes, i.e., synthesis/transport of lipids, Ca^2+^ dynamics/signaling, autophagy, mitochondrial shape and size, apoptosis, and energy metabolism [163]. MAMs are altered in neurological disorders such as AD, PD, and ALS [164]. Mitochondria acts as a hub of ATGs, supplying membranes for the formation of autophagosomes, and modulating autophagic flux [165]. Mitochondria also suffer UPR(mt), and depending on the activated pathway, it has been linked with extended lifespan in worms and mice [166], but its overactivation causes neurodegeneration [167].

Mitochondrial dysfunction arises from an inadequate number of mitochondria, an inability to provide necessary substrates to them, or a dysfunction in their electron transport and ATP-synthesis machinery. The high levels of ROS and the related reactive species (RNS) can be neutralized by dismutase enzymes and antioxidants [168]. Alterations in these enzymes and certain mitochondrial respiratory complexes have been observed in neurodegenerative diseases such as ALS and PD [169]. Perturbations in mitochondrial number and function severely impair cellular homeostasis and trigger the onset of disease. Therefore, cells pursue to maintain a dynamic balance between the opposing processes of mitochondrial biogenesis and clearance. The accumulation of dysfunctional mitochondria and/or the loss of its biogenesis produces cell death. Recent therapeutic avenues to prevent neurodegeneration aim to boost mitochondrial biogenesis by modulating NAD^+^ [170], epigenetic marks [171], or modulating the serotonin axis in the brain [172]. Dysfunctional mitochondrial clearance by mitophagy also yields neuroprotection. The overexpression of PTEN-induced kinase 1 (PINK1), which is essential for initiating the mitophagy process, increases neuronal survival in a fly model of HD [45]. In addition, NAD^+^ supplementation reduces neurotoxicity in a PINK1-mutant model of PD [173].

Mitochondria function is crosslinked with ROS and cellular antioxidant response. In that way, the transcription factor Nuclear factor erythroid-derived factor 2-related factor 2 (Nrf2) regulates the expression of cytoprotective and detoxifying genes to combat oxidative stress and neuroinflammation, aiming to reduce neural damage. Therefore, it can be an effective manipulation to delay disease progression in neurodegenerative diseases [174,175,176]. Under the stimulation of ROS, Nrf2 dissociates from Kelch-like ECH-associated protein (Keap1), thereby regulating the expression of antioxidant enzymes [177]. It has been described that Keap1 mediates the ubiquitination of p62 [178]. When Keap1 is downregulated, p62 is accumulated in cells and causes cytotoxicity, while its overexpression promotes the degradation of p62 via the autophagy pathway. On the other side, p62 activates Nrf2 through the autophagy pathway to form the p62-Keap1-Nrf2-antioxidant responsive element (ARE) pathway and counteracts the oxidative damage caused by ROS [179]. Moreover, Nrf2 forms regulatory loops involved in the regulation of mitochondrial biogenesis. Nrf2 increases the expression of peroxisome proliferator-activated receptor-gamma coactivator 1-alpha (PGC-1α) and nuclear respiratory factor (NRF1), which are directly involved in the regulation of mtDNA transcription. Lastly, Nrf2 regulates the expression of PINK1, which plays a key role in mitophagy induction [180], suggesting that the antioxidant capacity of the cell also impacts on mitochondria state.

Neurodegenerative diseases are related to both the inhibition of the Nrf2 pathway and dysfunction of autophagy, which leads to the accumulation of ROS, senescent organelles, and misfolded proteins [181,182]. Neurodegenerative diseases are related to lots of protein aggregates and ROS, inducing the p62-Keap1-Nrf2 positive feedback axis, which is a protective mechanism in neurons [183,184]. Nrf2 expression is low in AD animal models and AD patient brains [185]. Nrf2 binding to the ARE occurs soon during disease progression, which corresponds with an increase in ROS production [186]. Nrf2 neuroprotects by decreasing ROS generation and Aβ-mediated ROS-induced toxicity [187,188]. In HD, there is a dysfunction of the mitochondrial complex II, causing an increase in ROS [48]. In the initial phase of HD, the treatment with an Nrf2 agonist leads to an increase of vital cytoprotective genes via the Keap1–Nrf2–ARE in astrocytes and microglia [189]. Activation of the Keap1–Nrf2–ARE pathway by small molecules in astrocytes accelerates the resistance of neurons to the non-excitotoxic glutamate toxicity [46,47,48]. Altered mitochondria function, biogenesis, and mitophagy are important pathological features in PD, and Nrf2 is an important transcription factor that regulates mitochondrial quality control and homeostasis [190]. In PD, there is an activation of the Nrf2–ARE system [191,192] and its pharmacological activation prevents PD progression [49,50]. Nrf2 activation plays a protective role against ROS and cell death caused by the superoxide dismutase 1 (SOD1) mutant protein. In addition, the astrocyte Nrf2 overexpression increases the survival of the SC MNs and extends lifespan in SOD1 transgenic mice [51,52]. Besides, the crosstalk between p62 and the Keap1–Nrf2 pathway in the context of autophagy may play an important role in the removal of ROS, preventing oxidative damage and modulating of ER stress during cerebral ischemia-reperfusion injury [53].

Lastly, mitochondria drives neuronal survival, because they sense internal and external death initiators, triggering signaling cascades that converge at the mitochondria and then re-diverge into one or more cell death pathways that lead to a different type of cell death (such as intrinsic apoptosis) [193].

## 4. Targeting Systemic Modulation

### 4.1. Caloric Restriction

Caloric restriction (CR) extends lifespan in different organisms and has protective effects on several organs. CR impacts the whole organism: from systemic milieu to different subcellular populations. In 2010, Kromer and collaborators suggested that CR benefits are dependent on SIRT1-dependent autophagy [194]. On the other side, it has been pointed out that CR is neuroprotective in PD disease by a Ghrelin-AMPK axis, with AMPK being a key inducer of autophagy [195]. Given the obvious impossibility to maintain a long-term CR, therapeutic interest was raised to discover novel CR “mimetics” (CRM), which mimic the physiological effects of CR in the organism [196]. Both CR and CR-mimetics have probed efficacy in AD rat models by improving cognitive function through autophagy induction [197], so they are novel therapeutic avenues for treating neurodegeneration.

### 4.2. Exercise

Physical exercise is gaining interest due to its ability to reduce pathophysiological conditions such as neuropathic pain or improve functional outcomes in stroke models [198]. It also slows-down PD progression by the inhibition of the inflammatory reaction and the enhancement of antioxidant balance [199]. It is described that exercise acts by increasing endogenous levels of neurotrophic factors [200,201]. Moreover, it modulates muscle hormone secretion, promoting protective effects in the brain, neurogenesis, and ameliorating brain aging [202]. In fact, it has been recently described that the same hormone, irisin, has a role in bone formation [203], indicating that exercise impacts the whole body.

## 5. Finding Effective Neuroprotectant: What There Is and Where We Go

Common hallmarks of neurodegenerative diseases are a non-correct activation of UPR, the accumulation of autophagic processes, a mitochondrial well-function failure, among others. Altogether, they will overwhelm neurons, provoking their demise. An effective neuroprotectant must correct these mechanisms by boosting the cell with a complete resilience to aging/insults. We need to completely modify the molecular network within the cell, pushing it towards a complete restoring of functions. Approved drugs such as Riluzole for ALS [204], or clinical trials on-going, like Rapamycin for ALS [204], Spermidine, and DH for AD [205,206], only target one of these degenerative processes, and the neuron is overwhelmed by the other ones. Although they may yield beneficial effects, we propose to find a genetic or pharmacological approach to endorse different molecular pathways—multitarget therapy—instead of only one target.

Specific overexpression of certain proteins such as SIRT1, BIP, and/or ATG5 facilitates neuronal survival after nerve injury, and they neuroprotect in neurodegenerative diseases. They mainly fine-tune UPR or autophagy networks. SIRT1 activation by the use of transgenic mice or viral vectors demonstrated protection in different neurodegenerative diseases such as ALS, AD, and HD [207,208,209] and also after nerve injury [55]. SIRT1 deacetylase activity endorses different endogenous mechanisms of protection: autophagy, modulates UPR by attenuating PERK, and increases ATF6 cleavage [23,210], has anti-apoptotic effects, and modulates AKT activity to inhibit anoikis [211,212]. Therefore, the precise modulation of it may enhance cellular resilience. From our recent study, we conclude that modulate SIRT1 deacetylase activity is an essential node for the molecular network to achieve cellular resilience [54,55]. Lastly, BIP overexpression protects against aggregates and induces autophagy and mitophagy [99], so its modulation is also an effective approach to cluster different neuroprotective pathways.

## 6. Concluding Remarks

Boosting endogenous mechanisms of neuroprotection opens exciting therapeutic avenues to treat neurodegenerative diseases or maintain tissue homeostasis after neurotrauma. Although this is an unexplored field nowadays, it may promote more effective biomedical results than blocking a concrete pathophysiological hallmark. Therefore, endorsing them by genetic, pharmacological, or systemic-modulation therapies can delay pathology progression and enhance functional recovery. The optimal therapeutic strategy must involve a concrete modulation of the endogenous mechanisms of protection to re-model the complete network and achieve protection.

## Figures and Tables

**Table 1:** Summary of the proteins involved for each endogenous mechanism of neuroprotection, including the molecular mechanism by which their effects are mediated.

Protein Involved	Pathology	Mechanism of Protection	References
		Autophagy	
**ATG5**	PD ^1^	ATG5 induces autophagy and it orchestrates the autophagosome elongation by forming the complex with ATG16L1-ATG12. ATG5 overexpression protects by increasing the autophagy flux.	[4]
	Axotomized MNs ^2^		[5]
**ATG7**	PD	ATG7 induces autophagy by facilitating the conjugation of the complexes ATG12-ATG5 and ATG5 -ATG16L1-ATG12, which facilitates autophagosome formation. ATG7 overexpression protects by increasing the autophagy flux.	[4]
**p62**	Fly models characterized by protein aggregates	p62 manages the charge load in the autophagosome and plays a key role in the late stages of autophagosome formation. p62 has a protective role via the autophagic degradation of polyglutamine protein oligomers.	[6]
**TFEB**	PD model	TFEB overexpression modulates a transcriptional network that is essential for lysosome biogenesis and function, and also for the maintenance of autophagy flux.	[7]
	AD ^3^ mice model		[8]
**Beclin1**	Basal conditions	Beclin1 is essential for autophagy initiation and nucleation and has anti-apoptotic abilities.	[9]
		**Unfolded protein response**	
**BIP**	PD model	BIP is an ER chaperone that is protective and controls protein folding and orchestrates UPR components. Overexpression of BiP protects by promoting the folding of misfolded proteins.	[10]
**PERK**	The activated cytosolic domain of PERK phosphorylates eIF2α, inhibiting translation and inducing the preferential translation of ATF4 mRNA, which will upregulate essential genes to restore cell homeostasis. Under prolonged ER stress, when the pro-adaptive UPR fails, PERK will trigger pro-apoptotic signals through activation of downstream CHOP, promoting apoptosis.		
	Intracerebral hemorrhage	PERK early activation after insult neuroprotects by restoring proteostasis.	[11]
	Tau pathology	Overexpression or pharmacological activation of PERK has protective effects.	[12]
	Scrambled mice model and AD	Avert PERK activation diminishes neuronal death and improves age-related memory.	[13,14]
	Prion-disease in vivo model	Specific inhibition of PERK in astrocytes delays neuronal loss.	[15]
**CHOP**	Optic nerve crush model	CHOP has been linked to apoptosis after ER stress in multiple disease models. Deletion of CHOP promotes retinal ganglion cells’ survival.	[16]
**IRE1a**	Liver failure	IRE1α is an ER transmembrane protein that plays a major role in the initiation of the UPR both in mammals and lower eukaryotes. The activated cytosolic domain of IRE1 cleaves the 26bp intron from its substrate Xbp1, facilitating its translation to form the transcription factor Xbp1, which activates the transcription of UPR genes.	[17]
**Xbp1**	Xbp1 is a highly active transcription factor, which promotes the expression of ER chaperones and proteins involved in the ERAD.		
	Ischemia/reperfusion in heart	Xbp1 activation promotes cardiac protection.	[18]
	AD, PD and after stroke	Xbp1 activation promotes neuroprotection.	[19,20,21]
	Diabetic and ischemia-induced retinopathy	Protective effect through activation of XBP1-mediated UPR activation and inhibition of NF-κB activation.	[22]
	Optic nerve crush model	Xbp1 overexpression increases neuron survival.	[16]
**ATF6**	ATF6 mediates ER proteostasis by changing the transcriptomic network of the cell.		
	Nerve injury	Increase the cleavage of ATF6 is a neuroprotective modulation of the UPR.	[23]
	Ischemia models	Pharmacological ATF6 activation induces UPR.	[24]
	Stroke	ATF6 forced expression induces autophagy.	[25]
**ATF5**	Human epilepsy	ATF5 is selectively translated upon endoplasmic reticulum stress response in non-neuronal cells, and it is highly expressed in the developing brain, where it modulates the proliferation of neural progenitor cells. Adult neurons increase ATF5 levels under ER stress as a pro-survival mechanism.	[26]
		**Anti-apoptosis**	
**IAPs**	IAP proteins suppress apoptosis through the inhibition of caspases activation.		
	Ischemia model	IAPs activation mediates the GDNF pro-survival effects on MNs after target deprivation	[27]
	Neonatal nerve axotomy	Apoptotic death of MNs after target deprivation.	[28]
	Adulthood axotomy	IAPs overexpression blocks neuronal death.	[29]
	Ischemia	Ischemic preconditioning enables IAP to halt the caspase death cascade and thus prevents neuronal death.	[30]
**AKT**	Basal conditions	AKT is a pro-survival player by blocking apoptosis.	[31]
	Basal conditions	AKT inhibits p53-induced apoptosis.	[32,33,34]
	Basal conditions	AKT phosphorylates FOXO increasing cell survival.	[35]
		**Anti-anoikis**	
**PI3K/AKT**	Basal conditions	PI3K/AKT promotes the survival of the differentiated cells.	[36,37]
**MMP9**	MMPs degrade the main components of the extracellular matrix.		
	Cerebral ischemia	MMP9 inhibition reduces laminin degradation during ischemia.	[38]
	ALS ^4^ mice model	MMP9 inhibition protects the motor unit.	[39,40]
	AD models	MMP9 inhibition protects the motor unit.	[41]
		**Cytoskeleton**	
**KIF5c**	Excitotoxicity in vitro model	KIF5c plays a major role in mitochondrial transport. KIF5c mediates neuroprotection by regulating mitochondrial function and maintaining good cellular health in front of death signals.	[42]
**DCTN1**	Osteoclast differentiation	DCTN1 is a component of the cytoplasmic motor protein dynein and is implicated in cytoskeleton assembly and organization. DCTN1 overexpression prevents apoptotic death.	[43]
**RILP**	Basal conditions	The dynein adaptor RILP integrates the processes of autophagosome biogenesis and the retrograde transport to control autophagic flux.	[44]
		**Mitochondria**	
**PINK1**	PINK1 is a molecular sensor that accumulates at the outer membrane of damaged mitochondria to recruit the E3 ubiquitin ligase Parkin and initiate mitophagy.		
	Fly model of HD ^5^	Increasing PINK1 levels improves mitochondrial integrity and promotes neuroprotection.	[45]
**Nrf2**	Regulates the expression of cytoprotective and detoxifying genes to combat oxidative stress and neuroinflammation, which reduces neural damage.		
	HD	The activation of the Keap1–Nrf2–ARE pathway by small molecules in astrocytes enhances the resistance of neurons to non-excitotoxic glutamate toxicity.	[46,47,48]
	PD	Nrf2 pharmacological activation prevents PD progression.	[49,50]
	SOD1 mutant model	Astrocyte-specific overexpression of Nrf2 improves the survival of the spinal cord motoneurons and extends mice lifespan.	[51,52]
	Cerebral ischemia–reperfusion injury	p62 and the Keap1–Nrf2 crosstalk removes ROS by autophagy, preventing oxidative damage and modulating ER stress.	[53]

^1^ Parkinson’s disease (PD), ^2^ motoneuron (MN), ^3^ Alzheimer’s disease (AD), ^4^ Amyotrophic lateral sclerosis (ALS), ^5^ Huntington’s disease (HD).

## Data Availability

No new data were created or analyzed in this study. Data sharing is not applicable to this article.
